# Mycobacterium tuberculosis protein Rv2652c enhances intracellular survival by inhibiting host immune responses

**DOI:** 10.1002/iid3.70012

**Published:** 2024-09-06

**Authors:** Jihong Li, Yafeng Dou

**Affiliations:** ^1^ Yichang Key Laboratory of Integrated Traditional Chinese and Western Medicine for the Prevention and Treatment of Immunological Diseases The Second People's Hospital of China Three Gorges University Yichang China; ^2^ Hubei Key Laboratory of Tumor Microenvironment and Immunotherapy China Three Gorges University Yichang China; ^3^ Department of Laboratory The Second People's Hospital of China Three Gorges University Yichang China

**Keywords:** host immune responses, intracellular survival, Mycobacterium tuberculosis, Rv2652c

## Abstract

**Backgrounds:**

*Mycobacterium tuberculosis* (*Mtb*), the pathogen responsible for tuberculosis, secretes a multitude of proteins that modulate the host's immune response to ensure its own persistence. The region of difference (RD) genes encoding proteins play key roles in TB immunity and pathogenesis. Nevertheless, the roles of the majority of RD‐encoded proteins remain to be elucidated.

**Objects:**

To elucidate the role of Rv2652c located in RD13 in Mtb on bacterial growth, bacterial survival, and host immune response.

**Methods:**

We constructed the strain MS_Rv2652c which over‐expresses *Mtb* RD‐encoding protein Rv2652c in *M. smegmatis* (MS), and compared it with the wild strain in the bacterial growth, bacterial survival, virulence of Rv2652c, and determined the effect of MS_Rv2652c on host immune response in macrophages.

**Results:**

Rv2652c protein is located at cell wall of MS_Rv2652c strain and also an integral component of the *Mtb* H37Rv cell wall. Rv2652c can enhance the resistance of recombinant MS to various stressors. Moreover, Rv2652c inhibits host proinflammatory responses via modulation of the NF‐κB pathway, thereby promoting *Mtb* survival in vitro and in vivo.

**Conclusion:**

Our data suggest that cell wall protein Rv2652c plays an important role in creating a favorable environment for bacterial survival by modulating host signals and could be established as a potential TB drug target.

## Graphical abstract

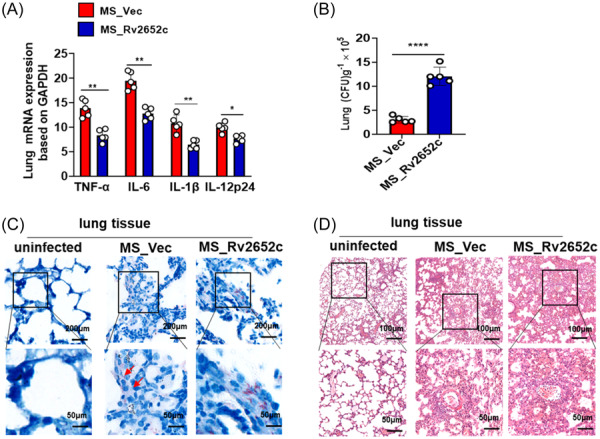

## 1 INTRODUCTION

Tuberculosis (TB) is a deadly infectious disease infected by *Mycobacterium tuberculosis* (*Mtb*) and poses a significant public health threat. According to the Global TB Report 2022 published by the World Health Organization (WHO), the COVID‐19 pandemic has undone previous progress in the field of TB treatment. In 2021, 10.6 million new TB patients and 1.6 million deaths were recorded.^1^ Currently, the lack of effective vaccines, ideal diagnostic techniques and the emergence of drug‐resistant TB pose serious challenges to TB prevention and control.^2^ Consequently, it is imperative to delve into the emerging virulence agents expressed by *Mtb* and to elucidate their underlying pathogenic mechanisms. Such investigations are crucial for combating tuberculosis and advancing its prevention and therapeutic strategies.

Comparative genomic analyses identified over 100 coding sequences absent from the BCG genome within genomic region differences (RD1 to RD16) that are present in the standard human *Mtb* strains H37Rv and pathogenic *M. bovis*.^3^, ^4^ Emerging research indicates that RDs could harbor novel functional antigens that are critical to the immune response and disease process in TB. Our previous studies have demonstrated that RD1 PPE68 and RD14 PE_PGRS31 facilitate the persistence of *Mtb* in macrophages and in murine models through the attenuation of inflammatory reactions.^5^, ^6^ To date, the role of most of the proteins encoded by RDs has yet to be deeply investigated. Therefore, there is an urgent need to study these proteins to enhance our understanding of the mechanisms of TB immunity.

The *Mtb* gene Rv2652c is 627 bp long and is located at RD13 region. Rv2652c may encode a phage terminase‐like protein. A previous study indicated that a prophage phiRv2 protein Rv2650c promote intracellular survival of mycobacterium in macrophages by inhibiting proinflammatory cytokines.^7^ Transcriptome analysis has revealed that the expression of Rv2652c is elevated in both *Mtb*‐infected macrophage models and under persisting conditions,^8^ implying that Rv2652c might serve as a regulator of the immune response and enhance *Mtb*'s ability to resist environmental challenges. However, a comprehensive elucidation of its contribution to mycobacterial infection remains to be fully explored.

To elucidate the function of Rv2652c, we engineered a recombinant strain utilizing the non‐pathogenic model strain *Mycobacterium smegmatis* (MS), designated as MS_Rv2652c. In this research, we demonstrated that Rv2652c enhances bacterial intracellular persistence and attenuates the release of proinflammatory cytokines both in vitro and in vivo. In addition, we have uncovered the effects of Rv2652c on signaling pathways that are involved in the production of proinflammatory cytokines.

## 2 RESULTS

### 2.1 Construction of the MS_Rv2652c recombinant strain

**Figure 1:**
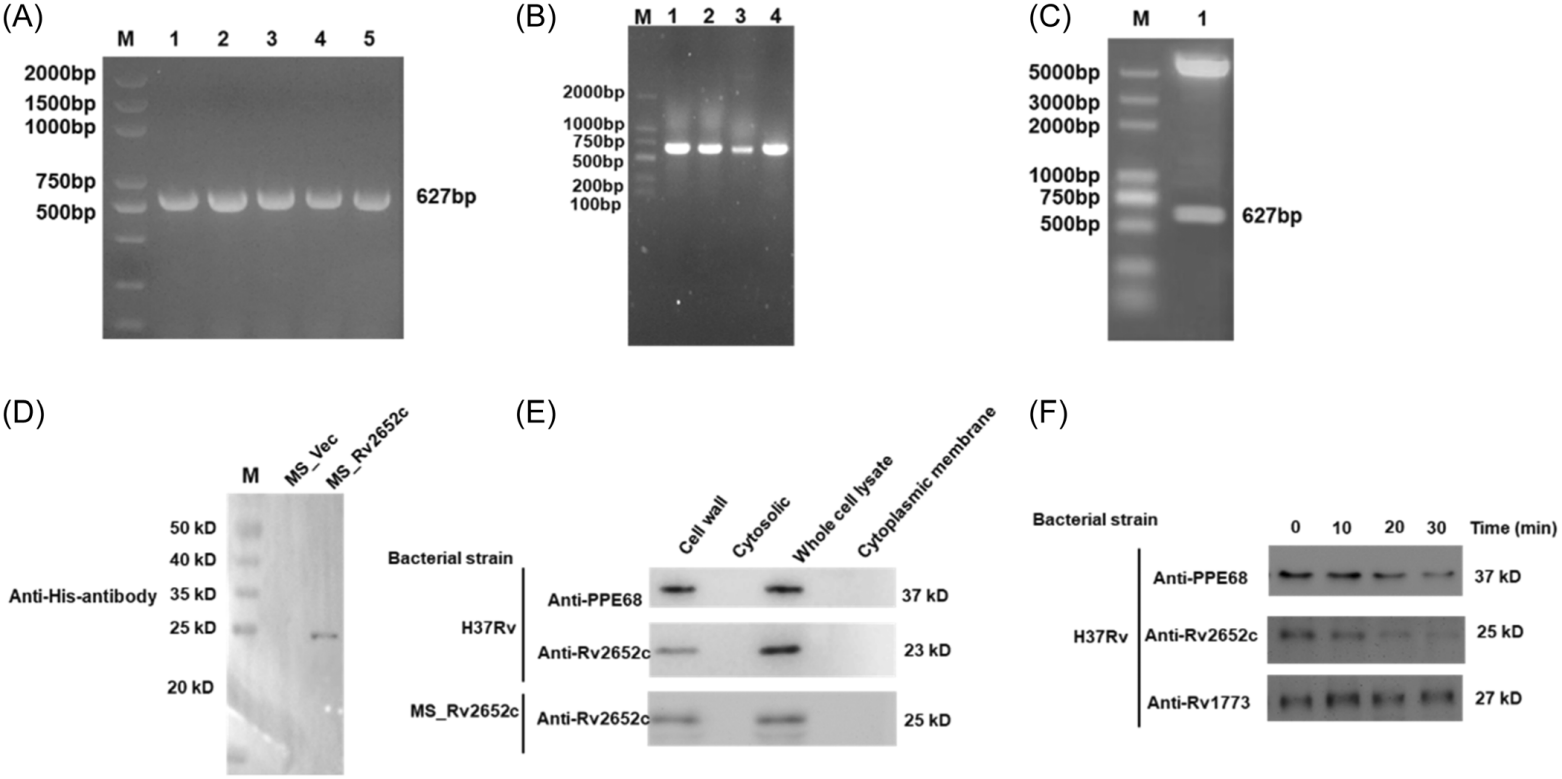
Rv2652c was identified as an integral component of the bacterial cell wall. (A) Agarose gel electrophoresis was performed to analyze the PCR amplification products of the Rv2652c gene using genomic DNA extracted from the H37Rv strain. M, DNA marker; 1‐5, product of Rv2652c gene by PCR amplification. (B) Recombinant plasmid pMV261‐Rv2652c was identified by colony PCR. M, DNA marker; 1‐4, product of Rv2652c gene by colony PCR amplification. (C) Recombinant plasmid pMV261‐Rv2652c digested with *BamH I* and *Hind III*. M, DNA marker; 1, pMV261‐Rv2652c digested with *BamH I* and *Hind III*. (D) Western blot analysis of Rv2652c protein of recombinant MS strains cell lysates using anti‐His antibody. M, protein marker. (E) Western blot analysis of subcellular localization of PPE68 and Rv2652c protein in H37Rv and recombinant MS_Rv2652c strain. (F) Western blot analysis of H37Rv lysates after proteinase K incubation. The cell wall protein PPE68 is a total cell wall control, Rv1773c is a cytosolic control.

The Rv2652c gene fragment of *Mtb* is approximately 627 bp in size. Genomic DNA from *Mtb* H37Rv was used as a template for PCR amplification of the Rv2652c gene (Figure 1A). To investigate the role of Rv2652c, we engineered two recombinant strains leveraging the MS mc^2^155 strain as a surrogate system for our research. Among them, MS_Rv2652c was able to express Rv2652c exogenously, while MS_Vec harbored the shuttle plasmid pMV261‐His, acting as the negative control. The recombinants were identified by PCR using specific primers that produced an amplicon of the anticipated 627 bp size (Figure 1B). Analytical restriction digest of pMV261‐Rv2652c plasmids using *BamH I* and *Hind III* were used to excise the 627 bp Rv2652c insert, which confirmed the successful cloning of the Rv2652c gene into the pMV261 vector (Figure 1C). Subsequent Western blot analysis analysis confirmed that the recombinant MS_Rv2652c strain had a molecular mass of approximately 25 kDa protein band (Figure 1D). Therefore, the reconstituted MS_Rv2652c strain was successfully created.

### 2.2 Rv2652c is associated with the bacterial cell wall

Western blot analysis was conducted to determine the subcellular localization of Rv2652c in recombinant MS, revealing a predominant presence of the Rv2652c protein in the cell wall (Figure 1E). It has been reported that PPE68 is present in the cell wall fraction of *Mtb* and Rv1773 is an intracellular protein.^9^, ^10^ After treatment of bacterial lysates with proteinase K, significant degradation of H37Rv bacterial cell wall proteins (PPE68 and Rv2652c) was observed in contrast to cytoplasmic proteins Rv1773 (Figure 1F). The findings imply that the Rv2652c protein is located at cell wall of MS_Rv2652c strain and also an integral component of the *Mtb* H37Rv cell wall.

### 2.3 Rv2652c enhances MS resistance to multiple types of harmful environments

**Figure 2:**
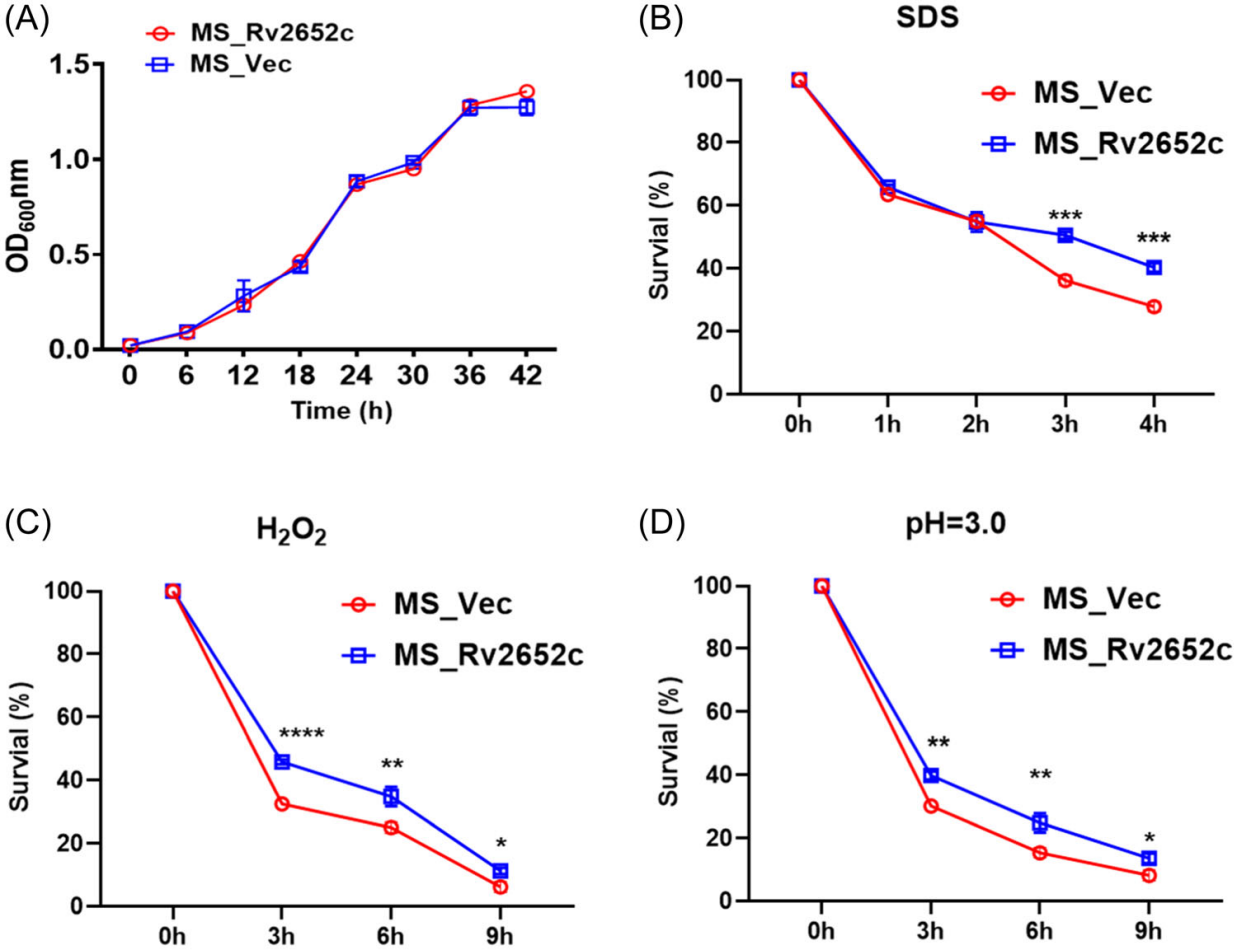
Effect of in vitro environmental stressors on growth of MS_Vec and MS_Rv2652c. (A) The growth curves of MS_Vec and MS_Rv2652c were monitored by measuring the OD_600_ every 3 h. (B–D) Survival of recombinant strains, MS_Vec and MS_Rv2652c, exposed to 7H9 liquid medium with 0.05% SDS (B), 5 mM H_2_O_2_ (C) and low pH (pH = 3.0) (D). Data are expressed as means of triplicate wells with ± SD. *p < .05, **p < .01, ***p < .001, ****p < .0001.

To investigate the role of Rv2652c, we conducted a growth profile analysis on the recombinant strains by monitoring the optical density at 600 nm (OD_600_ nm). The result indicated that there is no distinct growth difference between the recombinant strains, suggesting that the presence of Rv2652c does not alter the growth properties of MS (Figure 2A). *Mtb*, an intracellular pathogen, primarily invades macrophages and encounters various harsh conditions, such as nutrient scarcity, exposure to harmful chemicals, and oxidative stress. As explained in the method section, the recombinant strains were tested in adverse environments for their growth characteristics. Here, we observed that the survival rate of MS_Rv2652c survival was higher than MS_Vec upon exposure to 0.05% (w/v) SDS and 5 mM H_2_O_2_ (Figure 2B–C). Compared to the control strain MS_Vec, we also found that MS_Rv2652c had significantly higher survival rates in the acidic Middlebrook 7H9 (pH = 3.0) broth (Figure 2D). In summary, these findings strongly demonstrated that Rv2652c can enhance the resistance of recombinant MS to various stressors.

### 2.4 Rv2652c facilitates mycobacterial survival in macrophages

**Figure 3:**
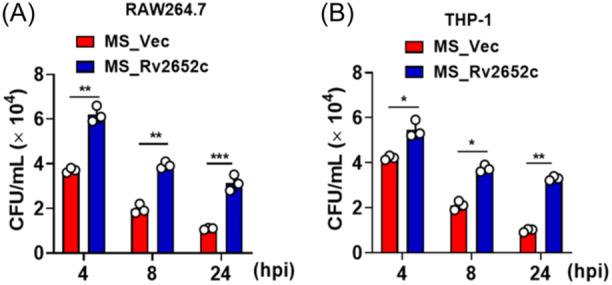
Rv2652c facilitate the survival of mycobacteria in macrophages (A, B) Bacterial CFUs were measured in RAW264.7 and THP‐1 cells infected with MS_Vec or MS_Rv2652c using a mixed infection experiment. Data are expressed as the mean ± SD, two tailed unpaired t test was used to determine statistical significance. *p < .05, **p < .01, ***p < .001.

To ascertain the influence of Rv2652c on the intracellular persistence of MS within macrophages, we initiated an infection with MS_Rv2652c and MS_Vec in RAW264.7 macrophages, following the protocols detailed in the methods section at a multiplicity of infection (MOI) of 10:1. There was no difference in the bacterial counts of MS_Vec and MS_Rv2652c phagocytosed in RAW264.7 cells (data not show). This suggests that Rv2652c does not contribute to enhancing the invasive capabilities of the recombinant MS_Rv2652c strain. After 2 h of phagocytosis (considered 0 h post infection), extracellular bacteria were eradicated using gentamicin and then bacterial survival was subsequently assessed through colony‐forming unit (CFU) enumeration at 24 and 48 h. Results showed that MS_Rv2652c exhibited a markedly increased intracellular survival rate when compared with MS_Vec (Figure 3A–B). The results suggest that the Rv2652c protein contributes to the enhanced persistence of MS within macrophages.

### 2.5 Rv2652c suppresses the generation of proinflammatory cytokines to modulate innate immunity

**Figure 4:**
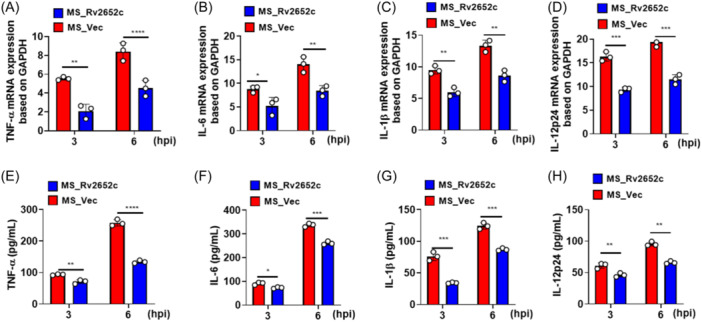
Rv2652c inhibits the host proinflammatory response. RAW264.7 macrophages were infected with recombinant MS_Vec or MS_Rv2652c strains with MOI of 10, respectively. The mRNA levels were analyzed by qPCR for TNF‐α (A) and IL‐6 (B), IL‐1β (C), IL‐12p24 (D) at the indicated time points. Culture supernatants from infected macrophages were harvested and detected for TNF‐α (E), IL‐6 (F), IL‐1β (G) and IL‐12p24 (H) production by ELISA. Data are expressed as the mean ± SD, two tailed unpaired t test was used to determine statistical significance. *p < .05, **p < .01, ***p < .001.

The proinflammatory immune response of the host regulates the outcome of infection.^11^ To explore the effect of Rv2652c on proinflammatory cytokines in macrophages, THP‐1 macrophages were infected with MS_Rv2652c and MS_Vec for 3 h and 6 h. The transcriptional and translational levels of proinflammatory cytokines (TNF‐α, IL‐6, IL‐1β, and IL‐12p24) were detected by RT‐PCR and ELISA, respectively. The results indicated a significant reduction in the mRNA expression of proinflammatory cytokines (TNF‐α, IL‐6, IL‐1β and IL‐12p24) in MS_Rv2652c at both 3 h and 6 h postinfection compared to MS_Vec (Figure 4A–D). Consistent with these results, the Rv2652c protein was shown to suppress the protein expression levels of TNF‐α, IL‐6, IL‐1β, and IL‐12p24 in MS‐infected THP‐1 macrophages (Figure 4E–H). In summary, Rv2652c markedly suppresses the secretion of proinflammatory cytokines in macrophages during MS infection.

### 2.6 Rv2652c inhibits the NF‐kB pathway activation during mycobacterial infection

**Figure 5:**
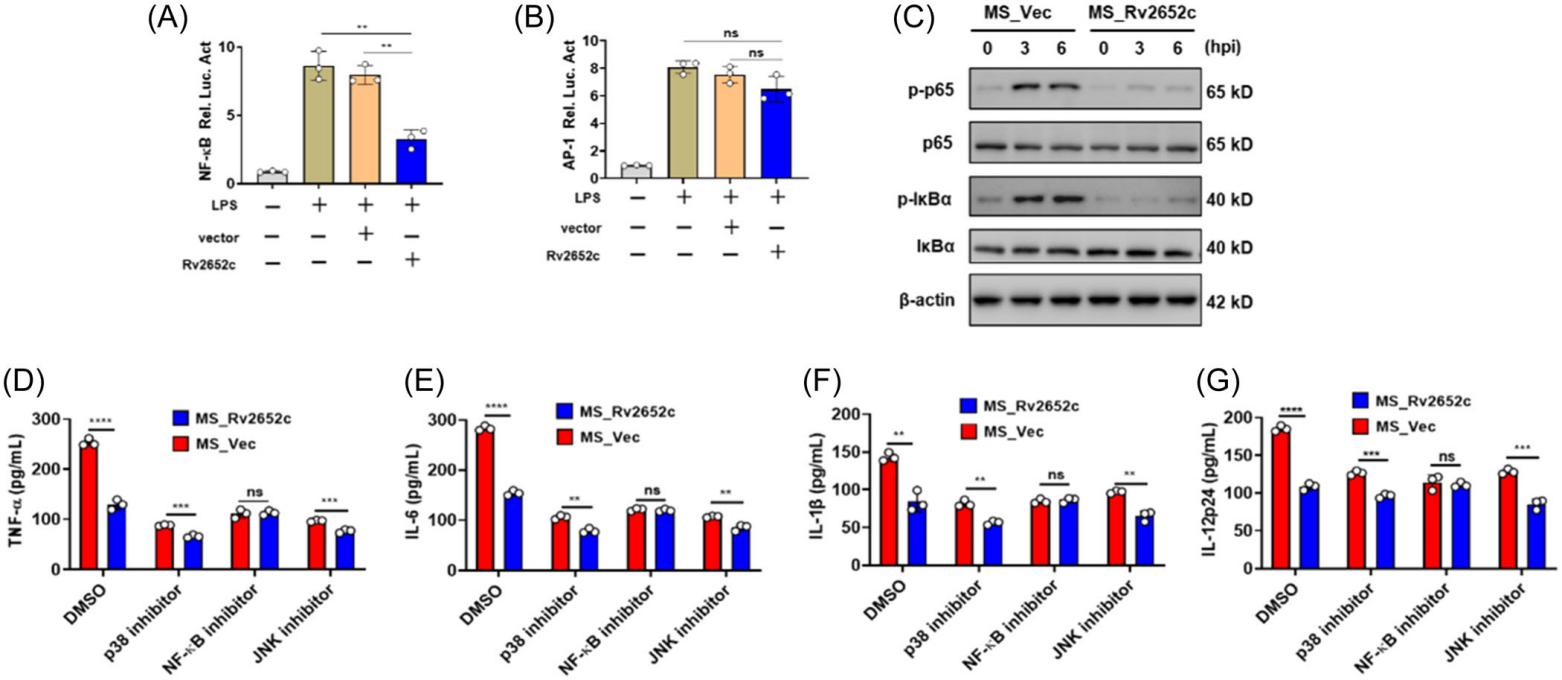
Rv2652c inhibits host inflammatory responses Rv2652c via suppressing NF‐κB signaling pathways. (A–B) The effects of Rv2652c on NF‐κB (A) and AP‐1 (B) signaling pathways were determined using a luciferase assay in RAW264.7 cells. (C) RAW264.7 cells were inoculated with MS_Vec and MS_Rv2652c (MOI = 10) at various time points. Western blot analysis of the phosphorylation levels of IκBα and p65. Quantification of the density of IκBα and p65 phosphorylated proteins relative to β‐actin. (D–G) Inhibitors of signaling pathways on MS‐induced inflammatory cytokines production. RAW264.7 cells were pretreated with DMSO, SP600125 (inhibitor of JNK), SB203580 (inhibitor of p38), or BAY11‐7082 (inhibitor of NF‐κB) for 24 h, and then infected with MS strains for 6 h. The culture supernatant was collected for measurement of TNF‐α (D), IL‐6 (E), IL‐1β (F), and IL‐12p24 (G). Data are expressed as the mean ± SD, two tailed unpaired t test was used to determine statistical significance; *p* > .05, not significant (ns); **p < .01, ***p < .001, ****p < .0001.

Following *Mtb* infection, the NF‐κB and MAPK signaling pathways are activated, leading to the production of inflammatory cytokines.^12^ To investigate the effects of Rv2652c on these signaling pathways, we analyzed its role in NF‐κB and AP‐1 luciferase activity in LPS‐stimulated macrophages. The results indicated that Rv2652c significantly inhibited NF‐κB luciferase activity induced by LPS while having no effect on AP‐1 luciferase activity levels (Figure 5A). We further detected the expression of phosphorylated p65, IκBα and total β‐actin (loading control throughout) in RAW264.7 infected with MS_Vec or MS_Rv2652c for the indicated time. The results of Western blot analysis indicated that MS_Rv2652c decreased the phosphorylation of p65 and IκBα compared to MS_Vec (Figure 5B). We then explored the potential role of NF‐κB pathway to the suppression of proinflammatory responses by Rv2652c using ELISA. Follow adding inhibitor of NF‐κB, no significant differences of the secreted proinflammatory cytokines (TNF‐α, IL‐6, IL‐1β, and IL‐12p24) of RAW264.7 were observed between the MS_Rv2652c and MS_Vec groups. However, there was also a significant difference in cytokine secretion between the MS_Rv2652c group and the MS_Vec‐infected cell group when p38 or JNK inhibitors were used (Figure 5C–F). These results suggest that Rv2652c inhibit MS‐induced generation of proinflammatory cytokines via modulation of the NF‐κB pathway.

### 2.7 Rv2652c suppresses inflammatory effects, promotes MS colonization and triggers lung injury in mice

**Figure 6:**
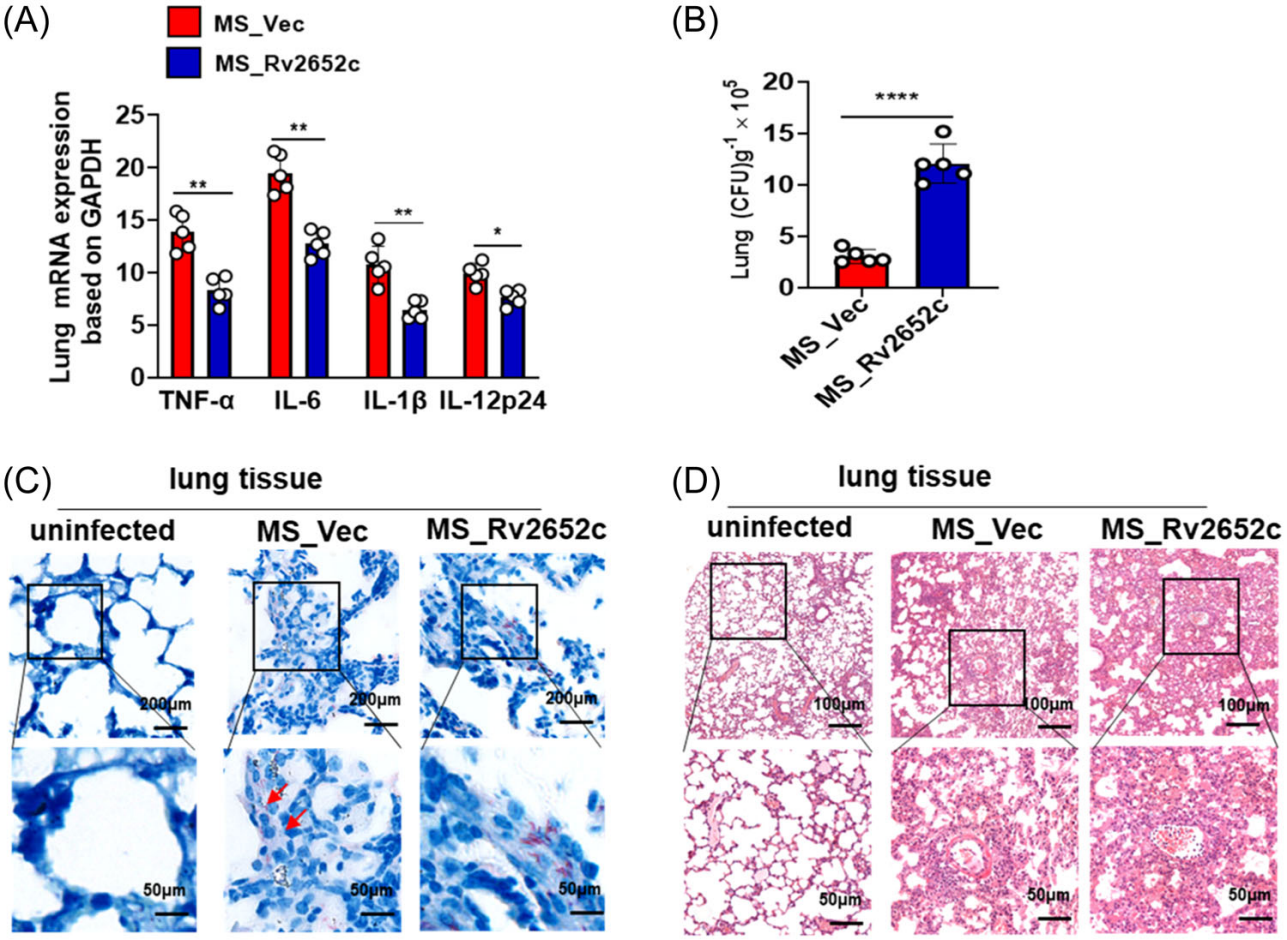
Rv2652c inhibits inflammation, facilitates MS colonization, and causes lung damage in mice. (A) RT‐qPCR was used to measure the mRNA expression of inflammatory cytokines TNF‐α, IL‐6, IL‐1β, and IL‐12p24 in the lungs of mice following infection with MS. (B) The colonization assay was conducted to quantify the bacterial colonies in the lungs of mice infected with MS. (C–D) The lungs of MS‐infected mice were subjected to acid‐fast staining (C) and H&E staining (D). Data are expressed as the mean ± SD, two tailed unpaired t test was used to determine statistical significance; *p < .05, **p < .01, ****p < .0001.

To determine the involvement of Rv2652c in the host's inflammatory and pathological injury in vivo, we utilized a mouse model of MS infection. Each C57BL/6 mouse was injected with 1 × 10^7^ CFUs MS via intranasal infection (*i.n.*). At 7 days postinfection, RT‐qPCR results indicated that the MS_Vec group had significantly elevated the expression of lung TNF‐α, IL‐6, IL‐1β, and IL‐12p24 compared to the MS_Rv2652c group (Figure 6A). MS_Rv2652c group had a significantly higher lung bacterial burden compared to MS_Vec group based on bacterial colony counting and acid‐fast staining (Figure 6B, C). Compared with the MS_Vec group, the MS_Rv2652c group demonstrated increased macrophage proliferation and granulomatous inflammation. Several alveolar occlusions and extensive inflammatory cell infiltration were seen in the lung tissues of mice infected with MS_Vec (Figure 6D). The findings suggest that Rv2652c inhibits host proinflammatory responses, thereby promoting *Mtb* survival and provokes inflammatory lung damage in vivo.

## 3 DISCUSSION

*Mtb* resides within the host cell, demonstrating proficiency in intracellular pathogenesis and harboring specific virulence factors that are absent in non‐pathogenic or mildly pathogenic mycobacteria. Research indicates that proteins encoded by genes in the RD region are closely linked to *Mtb* infection and immune evasion, and the absence of these genes has led to a notable decrease in *Mtb* virulence.^4^ For instance, EST12, a secreted protein found in the RD3 region, has been demonstrated to trigger localized macrophage death, stimulate the host's innate immune response, and impede early bacterial colonization.^13^ Furthermore, as RD region‐encoded proteins are exclusively found in virulent *Mtb* strains, diagnostics and vaccine development utilizing them possess unique advantages. Notably, ESAT6 and CFP‐10 from the RD1 region have been extensively employed in T‐SPOT diagnostics and vaccine research for TB.^14^ Presently, the functions of the majority of RD‐encoded proteins remain unknown. Therefore, investigating these proteins, which harbor unidentified functions, is not only conducive to understanding TB pathogenesis but also holds significant importance for the advancement of novel TB vaccines and diagnostic techniques.

In this research, we assessed the role of the prophage protein Rv2652c located at RD13 region in *Mtb*. We construct recombinant MS_Rv2652c to detect the resistance to multiple‐stressors, intracellular survival, cytokine responses. In the presence of acidity, oxidative stress, and surfactants, MS_Rv2652c was more resistant to macrophage infection than MS_Vec. And Rv2652c significantly promotes bacterial intracellular survival in vitro and in vivo. Further, RT‐qPCR and ELISA experiments confirmed that Rv2652c inhibits the expression of inflammatory factors TNF‐α, IL‐1β, IL‐6, and IL‐12p24 through the NF‐κB pathway. This study is the first to demonstrate the contribution of the prophage protein Rv2652c to the persistence of MS within macrophages.

Due to the presence and transcription of Rv2652c gene in *Mtb* lineages, but not in nonpathogenic bacteria, it is thought to play a role in virulence. In addition, transcriptomic analysis has shown that Rv2652c is upregulated in *Mtb*‐infected macrophages.^8^ Based on these results, Rv2652c appears to be a crucial protein that potentially enhances the adaptability of *Mtb* to environmental stress. In the phagosome, *Mtb* is exposed to acidic pH and reactive oxygen species such as H_2_O_2_. To circumvent host immune defenses, *Mtb* strategically utilizes its intrinsic elements or influences the host's cellular components to regulate the host's physiological processes and innate immune reactions, ensuring its survival within the host.^15^ The result from the Western blot analysis of the bacterial fractions indicated that Rv2652c is localized to the mycobacterial cell wall. In addition, further in vitro experiments demonstrated that Rv2652c significantly increased the intracellular survival capability of MS in macrophages and the tolerance to acidity and oxidative stress. There were no visible differences in the growth curves for MS_Rv2652c and MS_Vec, excluding the possibility that more bacterial cells assist MS in becoming more resistant to adverse conditions.

*Mtb* is an extremely adept intracellular pathogen with the ability to circumvent macrophage clearance mechanisms, thus enabling long‐term infections within macrophages.^16^ Cytokines are critical for the dynamic interaction between *Mtb* and the host.^17^, ^18^ The proinflammatory cytokine IL‐1β exhibits a dual role in *Mtb* infection.^19^ Recent studies have indicated that decreased levels of IL‐1β are associated with the development of severe tuberculosis symptoms.^20^ TNF‐α, a cytokine predominantly secreted by monocytes, macrophages, and dendritic cells upon infection with *Mtb*, is essential for granuloma formation and activates macrophages with immunoregulatory properties.^21^ IL‐6 plays a crucial role in the initial stages of the immune response to *Mtb* by driving the early production of proinflammatory cytokines, thereby fostering an environment conducive to the robust activation of T and B lymphocytes.^22^ IL‐12 plays an indispensable role in orchestrating the immune response to mycobacterial infections by promoting the differentiation of naive T cells into antigen‐specific Th1 cells, which are essential for pathogen clearance.^23^ Our findings reveal that the Rv2652c significantly suppresses the transcriptional and protein level of key proinflammatory cytokines, including IL‐6, IL‐1β, IL‐12p40, and TNF‐α. Consequently, we postulate that Rv2652c may exert immunomodulatory effects on inflammatory cytokines, thereby diminishing the bactericidal capacity of macrophages and facilitating the prolonged intracellular persistence of MS.

The critical role of MAPK and NF‐κB signaling cascades in modulating inflammatory responses during mycobacterial infections has been well‐documented.^12^ Various mycobacterial products have been found to activate different pathways. For example, Rv0927c has been found to modulate host immune responses by specifically suppressing proinflammatory cytokines through the inhibition of p38 MAPK and NF‐κB signaling cascades.^24^ On the other hand, Rv0222 has been demonstrated to subvert innate immune responses by targeting the JNK, p38 MAPK, and NF‐κB signaling pathways.^25^ Furthermore, Mce3E effectively inhibits the ERK1/2 signaling cascade, consequently suppressing the expression of proinflammatory cytokines.^26^ Our findings demonstrate that Rv2652c downregulates the expression of TNF‐α, IL‐6, IL‐1β, and IL‐12p24 by inhibiting NF‐κB pathways, leading to enhanced mycobacterial intracellular survival both in vivo and in vitro.

In summary, our study demonstrates that *Mtb* Rv2652c, a cell wall‐localized protein significantly suppresses the secretion of host proinflammatory cytokines, thereby fostering mycobacterial survival, which may lead to enhanced pathogenesis and tissue damage in the host. Our findings identified Rv2652c as a novel *Mtb* pathogenic factor that not only modulates the host's immune response but also represents a promising target for the development of new therapeutic strategies against TB. Further elucidation of Rv2652c's role in *Mtb* pathogenesis should involve studies using Rv2652c‐knockout mutants to dissect its specific contributions to virulence and immune evasion. As our lab lacked the required experimental environment to cultivate *Mtb*, fundamental investigations could only be conducted using model strains.

## 4 MATERIALS AND METHODS

### 4.1 Bacterial strains

*Mycobacterium tuberculosis* H37Rv (strain ATCC 27294) was obtained from Center for Wuhan University Animal Experiment/Animal Biosafety Level‐lll Laboratory. *M. smegmatis* (MS) mc^2^155 (strain ATCC 19420), *Escherichia coli* (*E. coli*) DH5α (strain ATCC 25922), and *E. coli* BL‐21 (strain ATCC BAA‐1025) were reserved in our laboratory. Mycobacterial strains were cultivated in Middlebrook 7H9 broth (7H9, BD Biosciences, Franklin Lakes, NJ, USA), supplemented with 0.05% (v/v) Tween 80, under continuous shaking, or on Middlebrook 7H10 (7H10, BD) agar enriched with 0.5% (v/v) glycerol. *E. coli* strains were growth in LB medium for DNA cloning and protein expression.

### 4.2 Animals and ethics statement

All artificial infection was carried out in strict accordance with the Guidelines for the Use and Care of Laboratory Animals in Hubei Province, China. These procedures were endorsed by the Ethics Committee of Three Gorges University under protocol number 2023070 A.

### 4.3 Cell culture

For the cell culture experiments, RAW264.7 cells were grown in DMEM supplemented with 10% FBS and 1% penicillin‐streptomycin. THP‐1 monocytes were differentiated into macrophages by suspending them in RPMI 1640 medium, plating at a density of 5 × 10^5^ cells per well in 12‐well plates, and treating with 20 ng/ml PMA for 24 h at 37°C in an atmosphere of 5% CO_2_, followed by adherence confirmation via light microscopy.

### 4.4 Plasmids, antibodies and reagents

In our study, the Rv2652c gene was PCR‐amplified from *Mtb* H37Rv genomic DNA and cloned into the pcDNA3.1‐Myc‐His vector for eukaryotic expression. Additionally, the pMV261 plasmid was employed to express Rv2652c in mycobacterial species. Both pGL3‐NF‐κB‐luc, pGL3‐AP‐1‐luc and Renilla were used in the Dual‐Luciferase Reporter Assay System. All primers used for cloning are listed in Table S1. Rabbit anti‐p65 (cat. no. 8242), rabbit anti‐p‐p65 (cat. no. 3033), rabbit anti‐p‐IκBα (cat. no. 2859) were all purchased from Cell Signaling Technology. β‐Actin mouse mAb (cat. no. AC004) was purchased from ABclonal Biotech. Anti‐Rv2652c, anti‐PPE68, and anti‐Rv1773 were rabbit polyclonal Abs prepared by our laboratory.^5^ SP600125 (JNK inhibitor), SB203580 (p38 inhibitor) and BAY11‐7082 (NF‐κB inhibitor) were purchased from MedChemExpress. TNF‐α, IL‐6, IL‐1β and IL‐12p24 were detected using ELISA kits (Dakewe Biotech, Beijing, China).

### 4.5 Construction of recombinant MS

The Rv2652c gene was expanded from the H37Rv genome and cloned into the pMV261 vector with *BamHI* and *HindIII* sites for multiple cloning. The resulting plasmid, pMV261‐His‐Rv2652c, was electroporated into MS. Recombinant MS strains were selected on 7H9 Middlebrook agar medium containing kanamycin, and positive clones were identified by colony PCR and confirmed by sequencing. The successfully transformed strains, MS_Rv2652c and MS_Vec, were subjected to Western blot analysis to detect the expression of the recombinant protein.

### 4.6 Subcellular localization of Rv2652c

The recombinant MS_Rv2652c and control MS_Vec strains were cultured in 7H9 broth medium. After 48 h of cultivation, bacteria were harvested and lysed by sonication. The lysates were centrifuged at 27,000 × g for 30 min at 4°C to separate the cell wall and cytosolic fractions. The presence of the recombinant His‐tagged Rv2652c protein in these fractions was assessed using SDS‐PAGE and Western blot analysis with an anti‐His mouse monoclonal antibody and an anti‐Rv2652c mouse polyclonal antibody, respectively.

### 4.7 Proteinase K sensitivity assay

Proteinase K sensitivity assay was conducted as previously described.^5^, ^27^ 8 × 10^9^ CFUs bacterial cells of H37Rv were cleaned with TBS buffer (Tris‐HCl pH 7.5, NaCl 150 mM, KCl 3 mM) and then cultured for 60 min at 4°C in the presence or absence of 100 mg/ml of Proteinase K (Sigma‐Aldrich). The reaction was terminated using a comprehensive cocktail of EDTA‐free inhibitors. The samples were then purified, resuspended, and analyzed via SDS‐PAGE and Western blot analysis employing mouse polyclonal antibodies targeting PPE68, Rv2652c, and Rv1773c.

### 4.8 Detection of in vitro survival under various stresses

Recombinant MS_Rv2652c and control MS_Vec strains were grown to the log phase before being exposed to stress conditions, including acidic pH (pH 3), 0.05% SDS, and 5 mM H_2_O_2_. All experiments were conducted in triplicate to ensure reliability. At each designated time point, the cultures were diluted and plated on MB 7H10 agar tablets. After a 3‐day incubation period, the number of CFUs was determined to assess bacterial survival under stress.

### 4.9 Mixed infection assay

THP‐1 or RAW264.7 cells were cultured in 6‐well plates and infected with MS_Vec and MS_Rv2652c at an MOI of 10:1 for 2 h at 37°C. After washing and gentamicin treatment, fresh FBS with hygromycin was added. Intracellular survival was assessed at 24 and 48 h by harvesting cells, washing in PBS, and lysing in Triton X‐100. The lysates were diluted and plated onto 7H10 agar for CFU determination at 37°C for 3–4 days. Survival rates were assessed by examining the intracellular bacterial counts at the beginning and during the sampling period, enabling the comparison of bacterial numbers over time to evaluate survival rates. All experiments were conducted three times to ensure reliability.

### 4.10 Measurement of cytokines

THP‐1 or RAW264.7 cells were infected with either MS_Rv2652c or MS_Vec strains for the predetermined durations. After 3 and 6 h, the total cellular RNA was extracted from infected cells by using an RNA extraction kit (TIANGEN, China). Next, cDNA synthesis was performed using the RevertAid First Strand cDNA Synthesis Kit (Thermo Scientific) and mRNA expression of cytokines (IL‐1β, IL‐6, IL‐12p40, and TNF‐α) were detected by quantitative real‐time PCR (qRT‐PCR) using gene specific PCR primers (Table 1). These qRT‐PCR reactions were performed on an ABI 7500 FAST Real‐Time PCR System using a QuantiFast SYBR Green PCR Kit (QIAGEN). Relative cytokine expression levels were determined after normalized to β‐actin expression. Culture supernatants were collected from the infected macrophages, concentrations of IL‐1β, IL‐6, IL‐12p40, and TNF‐α in the culture supernatants were determined using commercial ELISA kits according to the manufacturer's protocols (Dakewe).

**Table 1:** Primers list.

Primer names	Primer sequences (5'−3')
Rv2652c‐F Rv2652c‐R	GTGGATCCTTGCCATCGCCAGCAACC ATAAGCTTCCGGTCTGGGGCGAACGG
TNF‐α(mouse)‐F TNF‐α(mouse)‐R	AGGCACTCCCCCAAAAGATG CCACTTGGTGGTTTGTGAGTG
GAPDH (mouse)‐F GAPDH (mouse)‐R	ACCACAGTCCATGCCATCAC TCCACCACCCTGTTGCTGTA
IL‐6(mouse)‐F	TGTCTATACCACTTCACAAGTCGGAG
IL‐6(mouse)‐R	GCACAACTCTTTTCTCATTTCCAC
IL‐1β(mouse)‐F	GCCCATCCTCTGTGACTCA
IL‐1β(mouse)‐R	AGGCCACAGGTATTTTGTC
IL‐12p24(mouse)‐F	CGCAGCACTTCAGAATCACA
IL‐12p24(mouse)‐R	TCTCCCACAGGAGGTTTCTG

### 4.11 Dual‐luciferase reporter assay

The luciferase activities were detected using the Dual‐Luciferase® Reporter Assay System (Promega, Madison, United States), strictly adhering to the manufacturer's protocol for accurate and consistent results. pGL3‐NF‐κB‐luc or pGL3‐AP‐1‐luc, a Renilla luciferase reporter plasmid (pRL‐TK, Promega), pcDNA3.1‐Rv2652c or pcDNA3.1, were cotransfected into RAW264.7 cells. At 24 h posttransfection, the cells were subjected to lipopolysaccharide (LPS) treatment at a concentration of 1000 ng/ml (Sigma‐Aldrich) to induce the activation of NF‐κB and AP‐1 signaling pathways. Following a 1‐h LPS stimulation, cells were harvested, and luciferase activity was assessed. Luciferase data were standardized to account for transfection efficiency by determining the ratio of firefly to Renilla luciferase activity.

### 4.12 Mouse infection

In mouse infection assays, the bacterial cultures were disrupted into single‐cell suspensions using the BACspreaderTM 1100 sonicator (TB Healthcare, China) to ensure uniform dispersion before infection. C57BL/6 mice were purchased from the Center of Experimental Animals of China Three Gorge University. Mice were infected via intranasally (*i.n.*) with 1 × 10^7^ CFUs of MS_Rv2652c strain or the control MS_Vec strain, allowing for the comparison of the pathogenic effects of the Rv2652c gene. Lung tissues were collected and homogenized at 7 days postinfection and plating onto 7H10 agar plates. The plates were cultured for up to 3 days to allow for the growth of mycobacteria to determine the CFUs. Lung tissues were subjected to histological analysis with Ziehl–Neelsen acid‐fast staining and hematoxylin and eosin (H&E) staining to characterize the infection's pathological effects.

### 4.13 Statistical analysis

Unpaired two‐tailed Student's *t*‐tests and two‐way ANOVA followed by multiple comparisons were used for statistical analysis. The error bar represents the standard deviation (SD). Statistical analysis of quantitative data was conducted using GraphPad Prism 8.0. *p* values < 0.05 were considered to be statistically significant (*p < .05, **p < .01, ***p < .001, ****p < .0001).

## AUTHOR CONTRIBUTIONS

**Jihong Li**: Conceptualization; data curation; formal analysis; funding acquisition; investigation; methodology; software; validation; visualization; writing—original draft. **Yafeng Dou**: Funding acquisition; investigation; methodology; project administration; resources; supervision; writing—review and editing.

## CONFLICT OF INTEREST STATEMENT

The authors have no conflict of interest.

## ETHICS STATEMENT

Ethical approval was granted by the Ethics Committee of China Three Gorges University (2023070A).

## Supporting information

Supporting information.

## Data Availability

All raw data and code are available upon request.
