# Supplementary material for: Mycobacterium tuberculosis protein Rv2652c enhances intracellular survival by inhibiting host immune responses
Source: Immun Inflamm Dis. 2024 Sep 6;12(9):e70012. doi: 10.1002/iid3.70012 (PMC11378267; doi:10.1002/iid3.70012)
Supplement: Supplementary file 1 — Supporting information. [file IID3-12-e70012-s001.docx]

**Table S1 Primers used in this study**

| **Primer names** | **Primer sequences (5'-3')** |
| --- | --- |
| Rv2652c-F  Rv2652c-R | GTGGATCCTTGCCATCGCCAGCAACC ATAAGCTTCCGGTCTGGGGCGAACGG |
| TNF-α(mouse)-F  TNF-α(mouse)-R | AGGCACTCCCCCAAAAGATG  CCACTTGGTGGTTTGTGAGTG |
| GAPDH (mouse)-F  GAPDH (mouse)-R | ACCACAGTCCATGCCATCAC  TCCACCACCCTGTTGCTGTA |
| IL-6(mouse)-F | TGTCTATACCACTTCACAAGTCGGAG |
| IL-6(mouse)-R | GCACAACTCTTTTCTCATTTCCAC |
| IL-1β(mouse)-F | GCCCATCCTCTGTGACTCA |
| IL-1β(mouse)-R | AGGCCACAGGTATTTTGTC |
| IL-12p24(mouse)-F | CGCAGCACTTCAGAATCACA |
| IL-12p24(mouse)-R | TCTCCCACAGGAGGTTTCTG |
